# Insights into the inhibited form of the redox-sensitive SufE-like sulfur acceptor CsdE

**DOI:** 10.1371/journal.pone.0186286

**Published:** 2017-10-18

**Authors:** Esther Peña-Soler, Juan Aranda, Miguel López-Estepa, Sara Gómez, Fernando Garces, Miquel Coll, Francisco J. Fernández, Iñaki Tuñon, M. Cristina Vega

**Affiliations:** 1 Chemical and Physical Biology Department, Center for Biological Research (CIB-CSIC), Madrid, Spain; 2 Institut de Biologia Molecular de Barcelona (IBMB-CSIC), Barcelona, Spain; 3 Institute for Biomedical Research (IRB Barcelona), Barcelona, Spain; 4 Departamento de Química Física, Universitat de València, Burjassot, Spain; 5 The Scripps Research Institute, La Jolla, California, United States of America; 6 Abvance Biotech srl, Madrid, Spain; National Institute for Medical Research, Medical Research Council, London, UNITED KINGDOM

## Abstract

Sulfur trafficking in living organisms relies on transpersulfuration reactions consisting in the enzyme-catalyzed transfer of S atoms *via* activated persulfidic S across protein-protein interfaces. The recent elucidation of the mechanistic basis for transpersulfuration in the CsdA-CsdE model system has paved the way for a better understanding of its role under oxidative stress. Herein we present the crystal structure of the oxidized, inactivated CsdE dimer at 2.4 Å resolution. The structure sheds light into the activation of the Cys61 nucleophile on its way from a solvent-secluded position in free CsdE to a fully extended conformation in the persulfurated CsdA-CsdE complex. Molecular dynamics simulations of available CsdE structures allow to delineate the sequence of conformational changes underwent by CsdE and to pinpoint the key role played by the deprotonation of the Cys61 thiol. The low-energy subunit orientation in the disulfide-bridged CsdE dimer demonstrates the likely physiologic relevance of this oxidative dead-end form of CsdE, suggesting that CsdE could act as a redox sensor *in vivo*.

## Introduction

In living organisms the sulfur atoms needed to synthesize vital sulfur-containing biomolecules, including molybdopterin, biotin, thiamin, the heavily post-translationally modified tRNAs and the ubiquitous Fe-S clusters, are mobilized from L-cysteine as highly reactive persulfidic sulfur atom species [1,2] by PLP-dependent enzymes known as L-cysteine desulfurases (CDs; E.C. 2.8.1.7) [3,4]. The persulfide moiety is then transferred to desulfurase-specific sulfur acceptor proteins tasked with channeling sulfur down the appropriate pathway to its final destination. The highly interconnected network of proteins which collaborate to sustain sulfur trafficking in the model bacterium *Escherichia coli* comprises two large gene systems termed ISC (Iron-Sulfur Clusters) and SUF (Sulfur Mobilization) [5–8], which have been extensively characterized over the past two decades, and a third, smaller system, referred to as the CSD (Cysteine Sulfinate Desulfinase) system [9]. This interest has been fostered by the involvement of eukaryotic homologous proteins in human diseases such as Friedreich’s ataxia [10,11] and by the implications of these systems in the survival of pathogenic bacteria under oxidative conditions during infection [12,13]. In particular, the SUF system is upregulated under oxidative stress or iron starvation [14] to provide a backup system for the sustained trafficking of S from L-cysteine to, chiefly, essential Fe-S clusters, since those conditions suppress sulfur flux through the ISC system. Therefore, the SUF system acts as a safety net upon oxidative stress to avoid cellular obsolescence in the event that the ISC system collapses. It is then logical to ask what might be the consequences of oxidative stress conditions for the CSD system. The CSD genes, *csdA* and *csdE*, encode one CD (CsdA) and one sulfur acceptor (CsdE), which bear a significant degree of sequence similarity to the functionally equivalent SufS and SufE proteins (35% and 45%, respectively) encoded by the SUF system. Unlike SufE, CsdE becomes inactivated *in vivo* during cell extraction under non-reducing conditions, presumably by disulfide bridge formation through the only cysteine residue in the CsdE protein sequence (Cys61). Furthermore, a functional CsdA-CsdE system is necessary for ribosome translation efficiency and fidelity since the deletion of *csdA* or *csdE* causes the near complete loss of cyclic *N*^6^-threonylcarbamoyladenosine (ct^6^) modification at A37 of tRNA^ANN^ [15].

NMR and X-ray crystallographic methods have established the structural resemblance of CsdE and SufE. The first structure solved was that of *E*. *coli* SufE (PDB 1mzg) [16,17], shortly followed by those of the orthologous SufE proteins from *Thermus termophilus* HB8 (PDB 1wlo) (unpublished), *Salmonella typhimurium* (PDB 3g0m) (unpublished), and the BolA domain from *Arabidopsis thaliana* SufE1 (PDB 4pui) [18]. Several NMR and crystal structures have also been solved for *E*. *coli* CsdE. The first CsdE structure was that of the free (uncomplexed) sulfur acceptor determined by NMR (PDB 1ni7) [17]. A second free form of CsdE solved by X-ray crystallography was recently published (PDB 5eep) [19]. The remaining structures have been determined in complex with CsdE’s CD partner, CsdA, either in the unmodified or apo form (PDB 4lw4) [20] or in the doubly persulfurated state (PDB 5ft8) [21]. A hallmark feature of SufE and uncomplexed CsdE structures is that the reactive thiol is protected from solvent exposure into a hydrophobic cavity. This seclusion fulfills two functions. Firstly, the hydrophobic cavity hides the reactive thiol side-chain to avoid its spontaneous oxidation and therefore its waste. Secondly, the loop bearing the catalytic Cys is thought to be in a state of torsional stress, which poises it for a conformational change. Indeed, S transfer from the CD to the S acceptor is accomplished upon complex formation by a concerted structural rearrangement that results in the ejection of the catalytic Cys and the placement of its reactive thiol at a distance to the CD donor cysteine that is compatible with the transpersulfuration. In the CsdA-CsdE system, where this mechanism is better understood, CsdE undergoes drastic conformational changes leading to a swing-out motion of the hidden Cys61 by more than 11 Å and into the deep CsdA active-site groove. We and others have hypothesized that the efficient transfer of S atoms between SufS and SufE requires similar structural reorganizations to take place on SufE [20,21].

In order to shed light into the oxidation-induced inactivation of CsdE under oxidative stress, we have crystallized a disulfide-bridged dimer of CsdE, an inhibited species that was previously identified *in vivo* during protein purification [22]. This structure, together with that of the free CsdE monomer, was used for extensive molecular dynamics simulations that provided a detailed picture of the dynamical behavior of CsdE in the different states. The covalently linked dimer structure of CsdE provides new insights into CsdE susceptibility to self-inhibition under oxidative stress and reveals an intermediate state between the free and the CsdA-bound CsdE forms. The analysis of this intermediate state allows the description of the path followed by the reactive thiol from a fully solvent-protected conformation to a catalytically competent state, and informs about the structural plasticity and dynamics of CsdE and the closely related SufE.

## Materials and methods

### Protein expression and purification of native and SeMet CsdE

The procedure to express and purify *Escherichia coli* CsdE has been published previously [21]. Seleno-L-methionyl-labeled (SeMet) CsdE was produced by a metabolic inhibition protocol using a modified LeMaster minimal medium supplemented with 50 mg/ml SeMet in *E*. *coli* BL21(DE3) cells [23]. Native and SeMet CsdE proteins were purified using the same protocol except that 10 mM dithiothreitol (DTT) was added to all purification buffers for SeMet CsdE in order to avoid potential oxidation of selenomethionines. The typical yield of native and SeMet CsdE was 60 mg pure enzyme per liter culture.

### Crystallization of native and SeMet CsdE, X-ray data collection, structure determination and refinement

CsdE was concentrated to 40 mg/ml in a buffer containing 10 mM Tris-HCl, pH 8, 2 mM 2-mercaptoethanol (native) or 10 mM DTT (SeMet). Both native and SeMet CsdE crystallized in 0.1 M sodium citrate (pH 6.5), 20% (w/v) PEG monomethyl ether (PEG-MME) 2000 at 297 K and 0.2 M lithium sulfate, 23% (w/v) PEG 3350 respectively in sitting-drop vapor-diffusion experiments assembled with 1 μl CsdE and 1 μl reservoir condition. The crystals were flash-frozen in liquid nitrogen using 10% (v/v) glycerol as cryoprotectant. Complete data sets consisting of 360 images at 1.0°-oscillation angle were collected for SeMet CsdE crystals at the ID23-1 beamline (ESRF, Grenoble, France) [24] at the Se edge as judged from a fluorescence scan (0.97885 Å); and 180 images at 1.0°-oscillation angle were collected for native CsdE crystals at BL13-XALOC (ALBA, Barcelona, Spain) [25] at 0.97949 Å. The native CsdE crystals diffracted to 2.40-Å resolution and the SeMet CsdE to 2.95-Å resolution, both belonged to the *P*3_2_2_1_ trigonal space group, and both had two monomers in the asymmetric unit. The two data sets were processed with XDS [26] and scaled with Aimless [27]. Crystallographic statistics are given in S1 Table.

We used SAD phasing at the Se edge (0.97885 Å) to solve the structure of disulfide-bridged CsdE using the automated protocols built in the PHENIX suite of programs [28]. All of the 6 potential Se atoms were located and non-crystallographic symmetry (NCS) derived from the heavy atom positions was utilized during phase improvement (figure-of-merit 0.40) and automatic model building. Two hundred and twenty (220) amino-acid residues were placed (71% of the 308 possible residues) in electron density and were refined to R_free_/R_work_ of 0.26/0.28. This initial model was then used for phase extension into the native crystal at 2.40-Å resolution, and completed through cycles of maximum likelihood refinement in phenix.refine [29] interspersed with manual building and validation in *Coot* [30] and MolProbity [31]. The final model contains 283 amino-acid residues out of a total of 308 possible (residues 7−147 in chain A and residues 8−149 in chain B, where Lys148-His149 come from the purification tag) in two monomers, and 47 solvent molecules, with R_free_/R_work_ of 0.191/0.248, respectively. Refinement statistics are reported in S1 Table.

The coordinates and structure factors of CsdE have been deposited in the Protein Data Bank (PDB) with accession code 5nq6.

### Molecular dynamics simulations

#### Building the system

The initial coordinates for molecular dynamics (MD) simulations were selected from the disulfide-bridged CsdE structure described in this study (PDB 5ft7), using the complete covalent dimer. In addition, MD simulations of the free CsdE structure (PDB 5eep) [19] were performed. In either case, the p*K*_a_ of all titratable residues at pH 7.0 were calculated using Propka 3.1 [32] and hydrogen were added to the systems using the LEaP module of the AMBER program [33]. While the calculated p*K*_a_ of the Cys61 residue is 9.4, the previously published value for the experimentally determined p*K*_a_ was of 6.5 [22]. Therefore we carried out simulations for both the protonated and the unprotonated forms of the Cys61 residue within the free CsdE system. All systems were solvated with LEaP into a truncated octahedron box of TIP3P water molecules with a buffer of water molecules extending for 15 Å in every direction around the systems. A total of 1 or 2 Na^+^ cations were added to the respective systems to neutralize them. The final single-chain free CsdE systems consisted of 7781/7783 water molecules and 25,493/25,499 total atoms for the protonated/unprotonated Cys61 containing systems. The covalent CsdE dimer system consisted of 13,416 water molecules and 44,554 total atoms. MD simulations were conducted in AMBER 14 [34] making use of the PMEMD CUDA module with the TIP3P and AMBER14SB force fields [35].

#### MD simulations

We applied the same simulation protocol to the CsdE covalent dimer system, the free CsdE Cys61 protonated and unprotonated systems. Classical MD simulations were conducted with a time step of 2 fs and applying the SHAKE algorithm [36] to bond lengths involving hydrogen atoms. Simulations were carried out in the isothermal-isobaric ensemble with a pressure of 1 atm and a temperature of 300 K. The Berendsen algorithm [36] was applied to control the pressure and the temperature with a coupling constant of 5 ps. The Particle Mesh Ewald method [37] was used to compute long-range electrostatic interactions using standard defaults and a cutoff in the real-space of 10 Å. The systems were energy minimized, thermalized and pre-equilibrated for 10 ns before the production run was conducted. During this multi-step approach, we firstly equilibrated the water box, then released the side-chains gradually and finally released the backbone. A total time of 500 ns was simulated for the CsdE covalent dimer, and 1 μs for both the free CsdE protonated and the unprotonated system.

## Results and discussion

### Crystal structure of the disulfide-bridged CsdE dimer

**Fig 1:**
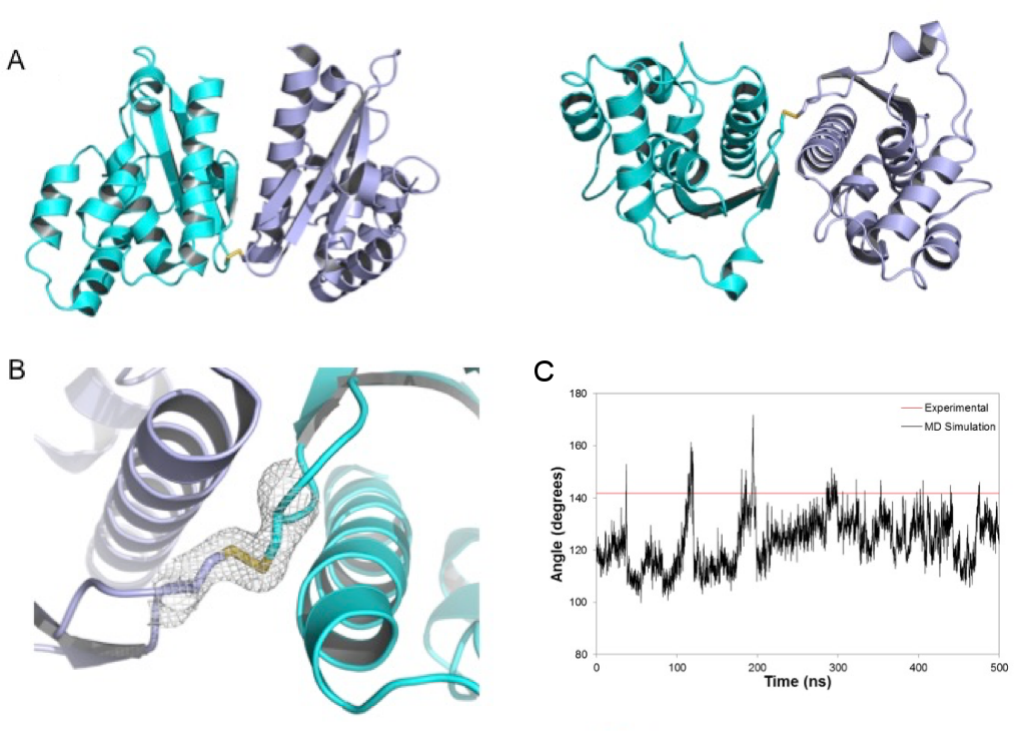
Crystal structure of the CsdE dimer. (A) Top (left) and side (right) views of the disulfide-bridged CsdE dimer, shown in cartoon representation with the two chains in cyan and slate blue colors. Cys61 side chain and the disulfide bridge between them is represented in sticks, with the sulfur atoms in yellow and the carbon atoms in chain colors. (B) Zoom-in into the disulfide bridge holding together the CsdE dimer. The electron density map is a σ_A_-weighted 2*DF*_O_−*mF*_C_ map contoured at 1.2 σ in grey. (C) Angle between the vectors joining the center-of-mass of the disulfide bridge and those of monomers of the CsdE dimer during the simulation. The red line indicates the value determined from the X-ray structure.

In order to shed light into the consequences of oxidation inactivation of the redox-sensitive sulfur acceptor CsdE, we determined the crystal structure of a disulfide-bridged CsdE dimer at 2.4 Å (Fig 1). We used single-wavelength anomalous diffraction at the selenium absorption edge (Se-SAD) to phase selenomethionyl-labeled CsdE crystals that diffracted to 2.95 Å, and performed phase extension in nearly isomorphous native CsdE crystals to 2.4 Å. The final model contains nearly all CsdE residues except for the first 7−8 amino acids, which were disordered. The R_free_/R_work_ converged to 0.167/0.238 at the end of the refinement protocol. Crystallographic statistics are reported in S1 Table.

The overall fold of CsdE in the covalently linked dimer is in agreement with that of the free CsdE, previously characterized by NMR (PDB 1ni7) [17] or X-ray crystallography (PDB 5eep) [19]. It consists of a two-layered α/β-sandwich with a central three-stranded antiparallel β-sheet (βA-βC), surrounded by seven α-helices (α1-α7); the reactive sulfur acceptor Cys61 is located at the tip of an extended β-hairpin between βA and βB (Fig 1). The disulfide bridge has a favorable stereochemistry and is well defined in electron density maps (Fig 1B). The root-mean-square displacement (rmsd) between the two CsdE protomers in the dimer is 1.09 Å (140 Cα atoms); the rmsd between the dimeric CsdE subunits and the crystal structure of free monomeric of CsdE is 0.83−0.88 Å (139−140 Cα atoms); and the rmsd with the NMR structure of CsdE is 1.40−1.67 Å (137−139 Cα atoms) (S2 Table). In the two (CsdA-CsdE)_2_ structures, a functionally significant conformational change is effected in CsdE [20,21]. In comparison with the apo (CsdA-CsdE)_2_, the rmsd between the free forms of CsdE and the disulfide-bridged dimer and the crystal structures of CsdA-bound CsdA is particularly large, especially in the persulfurated complex. The higher rmsd reflects the deeper conformational rearrangements underwent by the last two α-helices of free CsdE upon complex formation, with the partial unwinding of free-form α6 (α7 in complexed CsdE) and the split of α7 into α8’ and α8. In all comparisons, the greatest discrepancies occur around the catalytic Cys61. In the NMR and free-form X-ray CsdE structures, the side chain of Cys61 is buried inside a hydrophobic cavity that protects it from direct interaction with water molecules. However, in the disulfide-bridged CsdE structure this secluded orientation is obviously not possible; instead, the loop containing Cys61 has turned around to expose the thiol sulfur atom for disulfide bond formation. Interestingly, this exposed Cys61 conformation does not resemble the fully extended conformation observed for CsdA-bound CsdE, especially in the persulfurated complex, which is characterized by a swinging out motion where the thiol sulfur is displaced more than 10 Å. Indeed, the dimeric Cys61 conformation is roughly halfway between the fully protected and the completely exposed conformations, thereby representing an intermediate state. Given that this conformation does not create torsional stress around the Cys61-harboring loop and that there are no clashes, the crystallographic dimer is as stable as the free-form X-ray CsdE or the CsdA-bound CsdE conformations. Hence, this state might conceivably represent an intermediate step traversed by CsdE between the two extreme states (free and bound).

We conducted extensive molecular dynamics (MD) simulations of the disulfide-bridged CsdE dimer starting from the X-ray determined structure. The total simulation time for the dimer was 500 ns. The simulation of the CsdE dimer shows low rmsd values showing that no major conformational changes occur during our simulation. The most stable conformation that was sampled during the simulation corresponds to the structure observed in the crystal. We also measured the angle formed between the two centers of masses of the two CsdE monomers and the center of mass of the disulfide bridge during our simulation (Fig 1C). During the MD simulation, this intersubunit angle adopts an average value of 124° ± 9° (S1 Fig) close to the one determined from the X-ray structure (141º, Fig 1C and S2 Fig), showing that the latter structure does not present a significant torsional stress due to packing effects.

### Dimer interface of CsdE

Each CsdE molecule has a solvent-exposed area between 7210−7250 Å^2^ comprising 133−135 residues out of a total of 142 residues, according to PISA [38]. The crystal of dimeric CsdE is built from protein-protein interactions comprising distinct interfaces with buried surface areas in the range between 146−640 Å^2^, with 108−126 residues per subunit involved in interfaces. There are seven such interfaces in total, and the four largest interfaces involve contacts with crystallographic symmetry mates extending almost 2000 Å^2^ or 28% of a full monomer’s solvent-accessible area (ASA). The fifth largest interface, at 393 Å^2^ or merely 5.4% of a monomer’s ASA, includes the disulfide bond that creates this inactive CsdE species (S3 Table). The elongated shape and protruding Cys61-bearing loop help explain the modest size of this interaction surface, which is held together by the covalent linkage.

**Fig 2:**
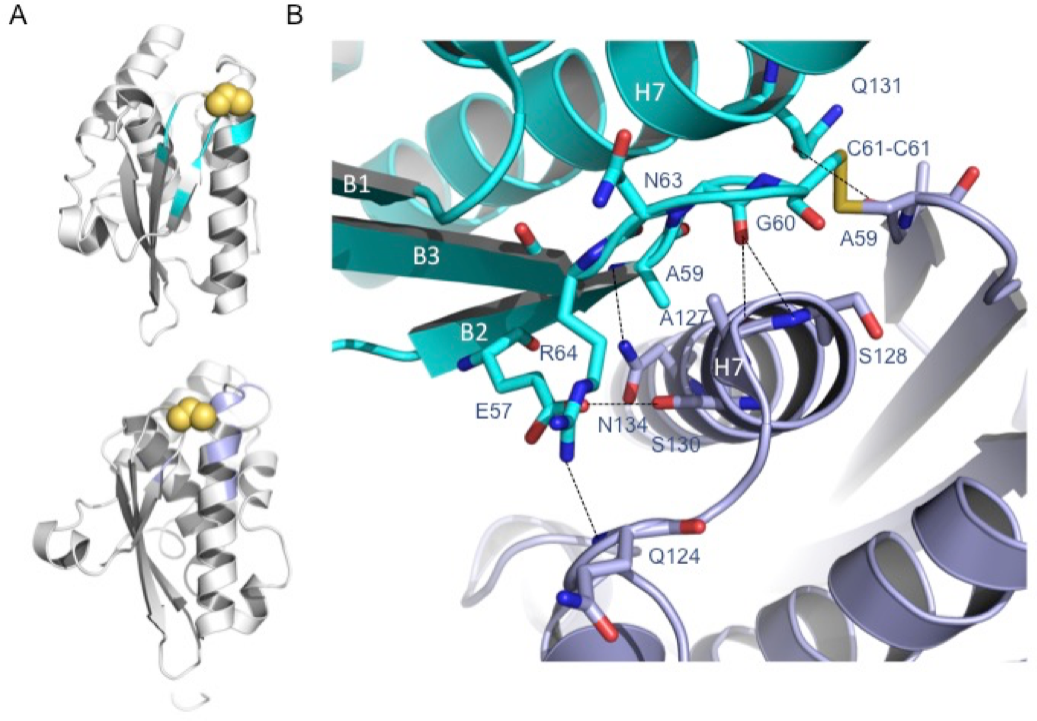
Interaction network at the CsdE dimer interface. (A) The two CsdE monomers are shown in ribbon representation. Cys61 side-chain atoms are shown in yellow to mark its position with respect to the monomer-monomer interfaces. The structural elements of each monomer contacted by the opposite monomer are shown mapped in slate blue (top) or cyan (below). (B) Close-up around the disulfide bridge depicting the details of the interaction network that consolidates the dimer. Helices α7 are labeled H7 and β-strands β1, β2, and β3 are labeled as B1, B2, and B3, respectively, only in the top monomer (cyan). Cys61 is harbored in the small loop connecting β2 and β3. Interacting amino acid residues are represented in sticks (main and side chains) and the interactions are depicted by black dashed lines.

**Fig 3:**
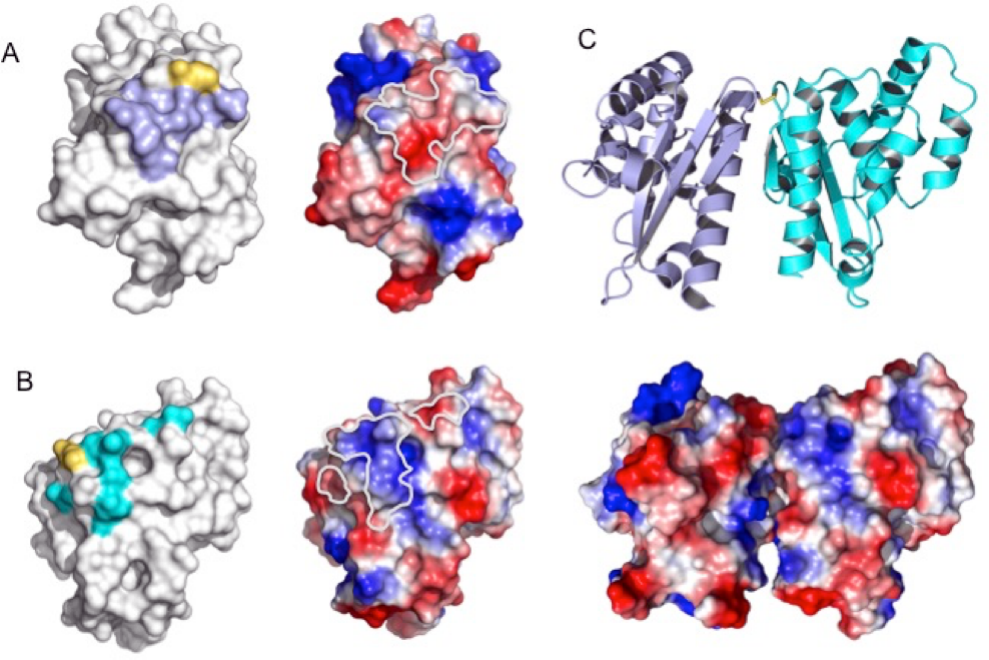
Electrostatic potential of interacting CsdE surfaces. (A, B) Pairs of molecular surfaces are shown with interaction surfaces (left) or electrostatic potential (right) mappings for each of the monomers. On the electrostatic potential surfaces, the positively and negatively charged areas are colored in blue and red, respectively; the outline of the interaction surface with the opposite monomer is drawn by a thick white curve. (C) Top view of the disulfide-bridged dimer (top) accompanying by the electrostatic potential surface (bottom) of the dimer, in the same orientation.

In the disulfide-bridged dimer, the two CsdE subunits are related by a pure rotation around an axis running parallel to the CsdE long molecular axis through 141° (S2 Fig). Since it is not a complete two-fold rotation, the two interacting faces of CsdE cannot be symmetric and the interactions mediated by the α7 helix are different on each monomer. Thus, the interacting residues in α7 on one of the CsdE subunits include the main-chain atoms of Ala127 and Ser128, the side chains of Ser130 and Asn134, and the side chain of Gln214 from the loop preceding α7 (Fig 2). In contrast, in the opposite subunit, only the side-chain carboxylate group of Glu57 interacts with the side chain of the opposite Ser130 in α7. The remaining interactions involve Arg64, Gly60, and Gln131 on non-α7 residues with other non-symmetric α7 residues (Cys61 and Gln124). The bonding through the disulfide bridge implies a closest interaction distance around Cys61 and makes it possible for Ala59 to be the only residue shared by both CsdE interacting surfaces. Despite its size, the intersubunit contacts involve highly electrostatically complementary surfaces, whereby the charge density at one side of Cys61 on βB and α7 on one subunit stabilizes the interaction with an oppositely charged surface patch made up by the βA-βB loop on the second CsdE subunit (Fig 3). The contour surrounding the interacting surface areas on either CsdE subunit clearly delineates areas of concentrated negative and positive charge that come close in the disulfide-bridged dimer.

We also used the MD simulation of the bridged CsdE dimer to analyze the contacts established between the two monomers. In particular, we measured the number of intersubunit hydrogen bonds, defined by a distance smaller than 3.2 Å between donor and acceptor atoms and an angle larger than 140° between the donor-hydrogen-acceptor atoms. We found two types of hydrogen bonds during the simulation: direct hydrogen bond interactions, where the donor and acceptor atoms belong to different monomers, and water-bridged hydrogen bond interactions, where a water molecule establishes simultaneously hydrogen bond interactions with two residues of different monomers. In agreement with the structural analysis of the X-ray structure, the number of direct hydrogen bonds between monomers is quite low (2.1 ± 1.4), although there are oscillations during the simulation reaching up to 7 contacts in some snapshots (S3 Fig). The contact between monomers is also stabilized by the presence of water molecules bridging between them. The average number of bridging waters was 4.4 ± 2.4, but it can reach values as large as 16 in some transient structures (S3 Fig).

### Cys61 as an interaction hub

**Fig 4:**
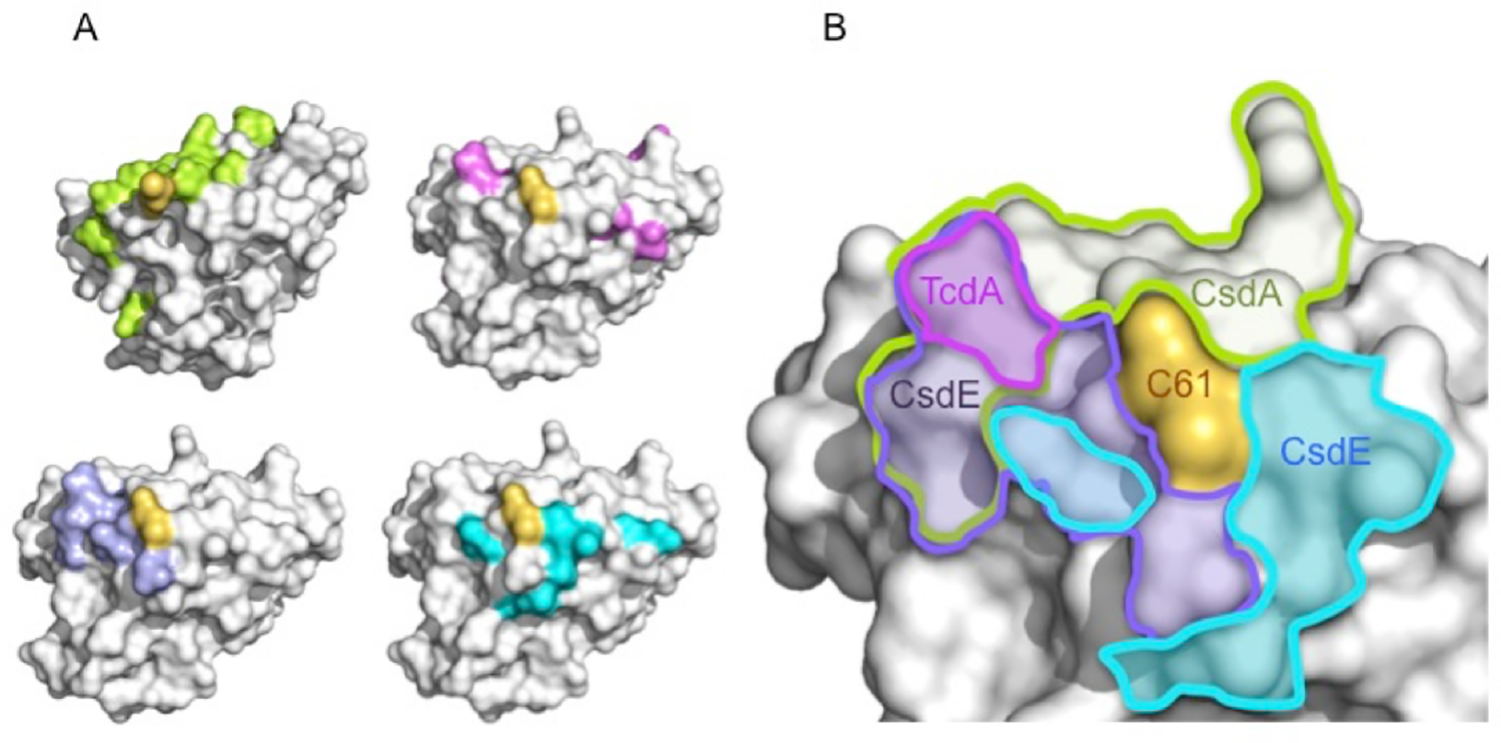
Cys61 as an interface hub on CsdE exposed surface. (A) Opaque molecular surfaces of CsdE (white) with the position of Cys61 mapped out in yellow. From the top right corner and following a clockwise rotation, the following interaction surfaces are shown: TcdA (in pink), CsdA (persulfurated complex, in green), the two non-symmetric CsdE interaction surfaces (in cyan and in blue slate). (B) Close-up on the molecular surface of CsdE around Cys61 (yellow; labeled C61). The outlines of the interaction surfaces shown in (A) are drawn in thick line with the same color code; each area is labeled with the protein that occupies the respective surface area.

Owing to the variety of macromolecular interactions that are possible for CsdE, we attempted to segment CsdE’s surface are according to each identified protein partner (Fig 4). We analyzed the molecular surfaces calculated for each crystal or NMR CsdE structure and mapped on those surfaces the residues that have been identified as important for the interaction (CsdA and CsdE) or that have been shown by NMR to be perturbed upon addition of a second component (TcdA) (Fig 4A). In all cases, the mapped Cys61 is depicted in yellow while the interaction patches for TcdA (purple), CsdA (green), or CsdE (cyan and slate blue) are shown in protein-specific colors. When the complete set of CsdE residues contacted by every partner is mapped on the same molecular surface, it is noticeable that Cys61 is the only residue shared by all partners and that the remaining residues cluster tightly around it (Fig 4B). While the CsdA-interacting residues are located on an extended band that curves around Cys61 (encircled in green), the TcdA-perturbed residues occupy a much smaller area (in purple) that overlaps with part of the CsdA-interaction region. Finally, the two non-symmetric areas engaged in the disulfide-bridged CsdE dimer appear to populate the opposite side of the CsdA and TcdA-interacting areas on either side of Cys61, with one of them partially overlapping with TcdA and CsdA (slate blue) and the other one characterizing a unique surface area patch (cyan). In conclusion, CsdE-interacting areas appear to have evolved around the functionally essential Cys61, occupying most residues in its immediate vicinity and therefore defining Cys61 as an interaction hub.

### A stepwise conformational path for CsdE Cys61 exposure

**Fig 5:**
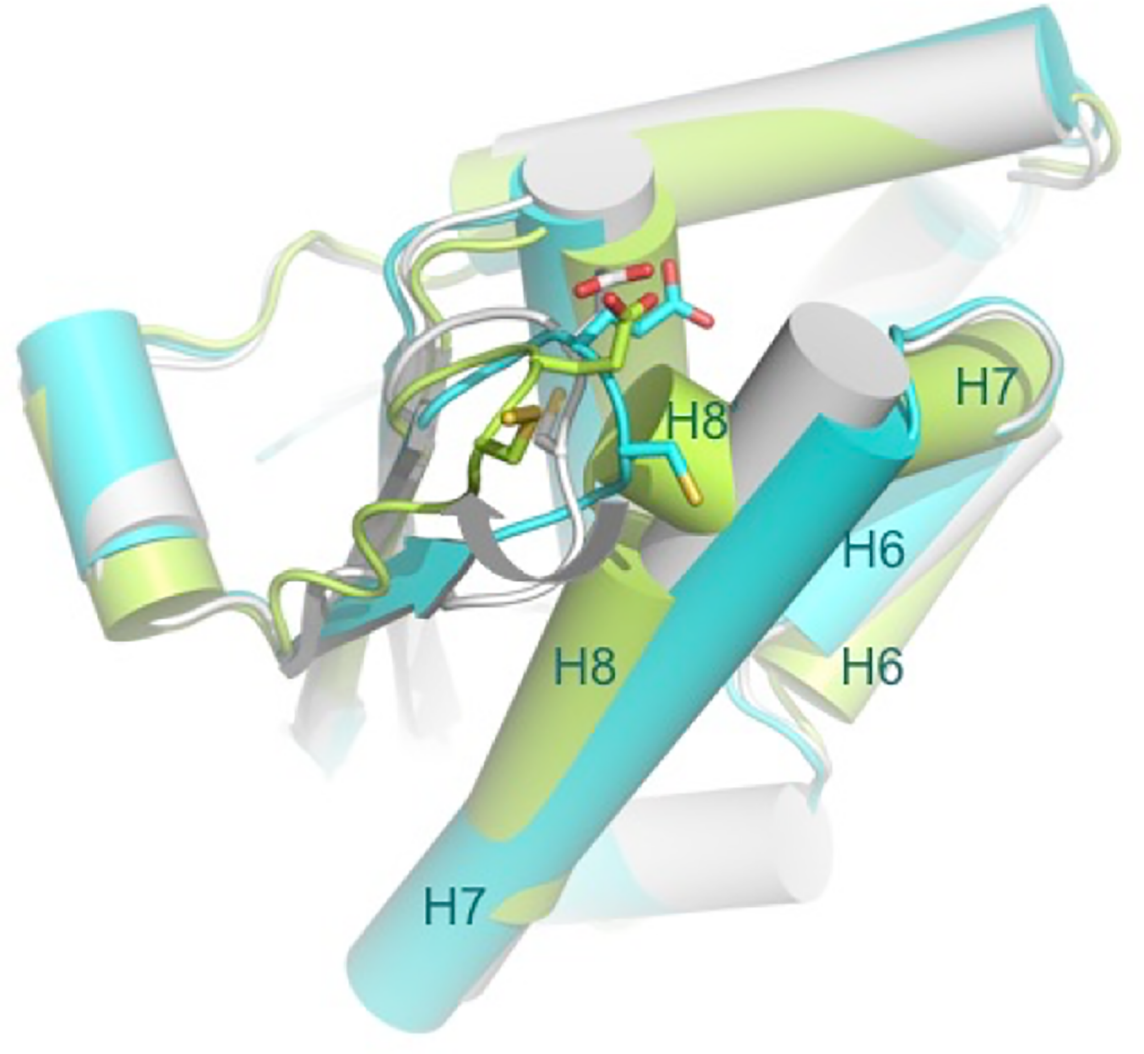
Trajectory followed by the Cys61 side chain of CsdE as it approaches CsdA for reaction. Three structures of CsdE are superimposed and represented in cartoons, with helices shown as cylinders. NMR CsdE is colored white, X-ray CsdE is in cyan, and the fully rearranged CsdE observed in the persulfurated (CsdA-CsdE)_2_ complex is in green. Helices α6, α7, and, for persulfurated CsdE, α8’ and α8, are labeled in order to facilitate comparisons. The side chains of Cys61 and Glu62 are shown in sticks with carbon colors according to the corresponding CsdE structure and sulfur/oxygen atoms in CPK colors.

The various available CsdE structures represent a comprehensive sampling of conformational states, from the free form of CsdE in solution (NMR CsdE) or in crystalline state (X-ray CsdE) to the deeply remodeled CsdE conformation described in the persulfurated (CsdA-CsdE)_2_ complex, through the intermediate states represented by the CsdE structures found in the disulfide-bridged CsdE dimer (Fig 5). Moving from the former to the latter, the Cys61 loop undergoes two superimposed movements; firstly, the demasking of Cys61 thiol function from the buried state in the free forms of CsdE to the completely exposed form epitomized by the persulfurated CsdA-bound CsdE; and, secondly, the swinging out of the Cys61 loop, which, accompanied by the rearrangements of the C-terminal helices, defines the trajectory followed by the reactive thiol group.

A remarkable observation made in the CsdE dimer is that, while Cys61 presents a fully exposed state as in persulfurated (CsdA-CadE)_2_, helix α7 is essentially unchanged with respect to the free forms of CsdE, and it is only slightly tilted to make way for the outward-going Cys61 loop (Fig 5). The co-existence of the C-terminal α7 helix structure and the exposed Cys61 conformation in the absence of CsdA suggests a dynamic scenario whereby CsdE could exist in two different states, with Cys61 either buried or partially exposed. In contrast, the equivalent residue in SufE (Cys51), which lies inside a hydrophobic cavity and is therefore occluded from solvent, is more resistant to oxidation than CsdE Cys61 or IscU Cys63/Cys106 when treated with challenging concentrations of hydrogen peroxide (H_2_O_2_) [39]. This is in agreement with the cellular function of SufE under oxidative stress, whereas under mild oxidative conditions, CsdE becomes locked in disulfide-bridged dimers [22]. The differential exposure of the reactive cysteine residues can account for the difference observed in their behavior with pro-oxidative molecules if we assume that SufE Cys51 is buried a greater fraction of time when compared with CsdE Cys61. The more hydrophobic nature of the cavity on the SufE surface that hosts Cys51 provides further evidence that this is a reasonable assumption.

**Fig 6:**
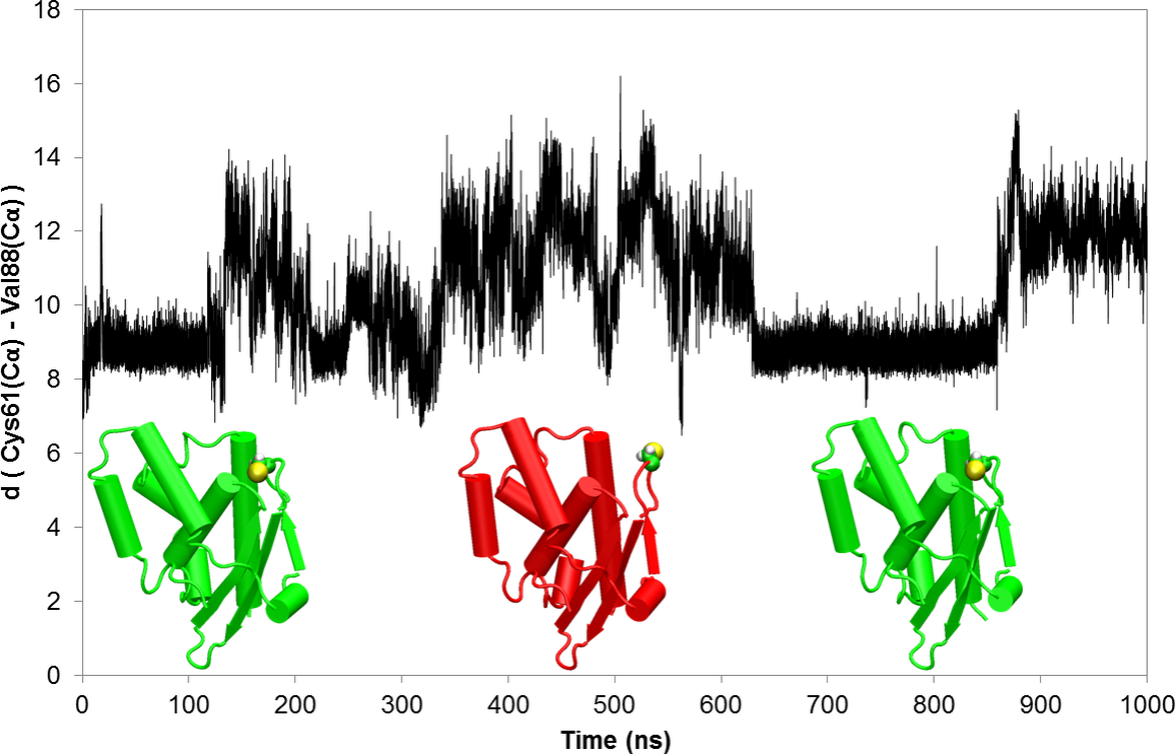
Distance between the Cα atoms of Cys61 and Val88 during the MD simulation of the CsdE monomer in solution with an anionic Cys61. Representative structures over the MD simulation are depicted, where the protein is colored in green for the buried conformation of Cys61 and in red for the solvent exposed conformation, Cys61 residue side chain is represented with balls.

Since the proposed equilibrium between buried and exposed conformations of CsdE Cys61 would not require major conformational changes in CsdE, they could be reasonably well populated under physiologic conditions. In this scenario, binding to CsdA would likely entail a selection of the partially exposed Cys61 conformation, which, then, would be further rearranged by interactions with CsdA to promote a better binding and facilitate catalysis. An alternative scenario can also be envisioned whereby an initial contact between CsdE with a buried Cys61 conformation would be followed by a progressive exposure of Cys61 following a path characterized by the discrete structural states previously described. In order to investigate whether this scenario was feasible, we conducted extensive MD simulations of the free CsdE monomer, both considering a protonated and an unprotonated state for the Cys61 residue. The total simulation time for each of the free CsdE monomers was 1 μs. The MD simulation of the free CsdE monomer with a protonated Cys61 residue showed low rmsd deviations (S4 Fig). We computed the *B*-factors from both simulations of CsdE to be compared to the experimental ones (S5 Fig). The computed *B*-factors reproduce the overall trend determined from the X-ray measurements. In the case of the simulation with a neutral Cys61, the *B*-factor of this residue is significantly smaller than the experimental value (S5A Fig), reflecting that the Cys61 in its protonated form is buried inside the protein pocket with low mobility. However, the analysis of the calculated *B*-factors obtained from the simulation of the free CsdE monomer with Cys61 in its anionic state shows that the mobility of this residue is highly increased (S5B Fig). This is due to a transition from being buried inside the pocket to a solvent exposed conformation captured in our MD simulation of free CsdE with unprotonated Cys61. Fig 6 shows the time evolution of the distance between the Cα atoms of Cys61 and Val88 as a measure of the conformational change of the Cys61 residue. Val88 is part of hydrophobic pocket where Cys61 is buried, in the loop located in front of the cysteine residue at a distance of 8.5 Å in the X-ray structure. During our simulations of free CsdE monomer with an unprotonated Cys61 the distance rapidly increases up to 14 Å (Fig 6), indicating the movement of Cys61 from being buried inside the pocket to a solvent exposed conformation. This movement implies no drastic conformational rearrangements of the α7 helix structure that remains essentially unchanged with respect to that found in free CsdE, with Cys61 buried in the hydrophobic pocket, and in agreement with the observed X-ray structures that trace the path from buried to exposed conformations.

**Fig 7:**
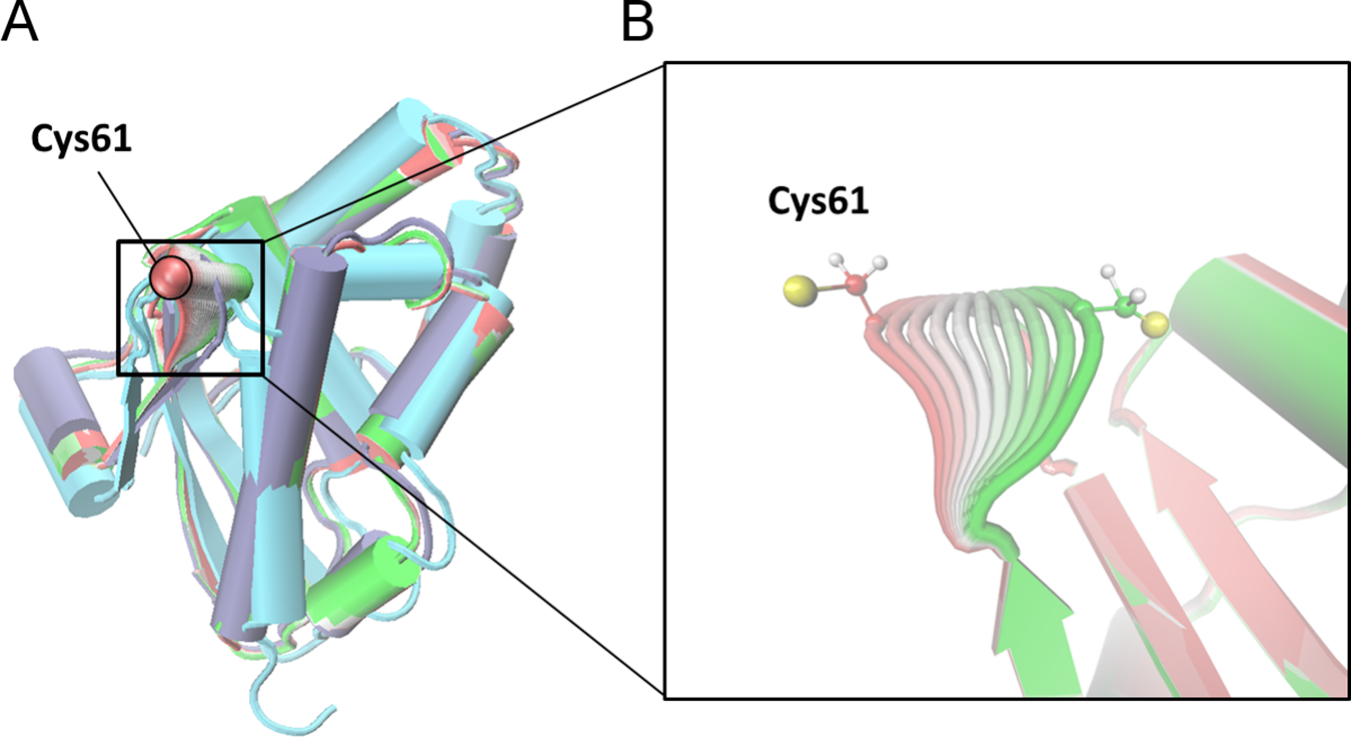
Structural comparison of CsdE along the proposed conformational change. (A) Superposition in cartoon with helices shown as cylinders of the CsdE free monomer from X-Ray (overlaid structures colored in green, white and red to highlight the movement, with the Cys61 Cα atom represented as a sphere), the CsdE monomer of dimer of the present study (pale blue) and CsdE monomer from the X-Ray structure of the (CsdA-CsdE)_2_ complex (cyan). The CsdE free monomer is depicted over the ensemble-weighted maximally correlated mode contributing to the change in the selected distance (d[Cys61(Cα)–Val88(Cα)]). (B) Insight of the CsdE free monomer’s movement of the loop is shown with Cys61 side chain represented as balls and sticks.

To further investigate the Cys61 movement we applied a partial least square functional mode analysis (PLS-FMA) [40] to our simulation in order to identify the collective mode of motion associated to the Cys61 displacement from buried to solvent-exposed. We used the distance between Cys61 and Val88 Cα atoms as the functional property measuring the buried/exposed transition. In our analysis, we considered all the backbone atoms of the CsdE monomer and used half of the trajectory as the model training set and the other half for cross-validation set. The PLS-FMA method converged at 10 components (S6 Fig), which capture the collective motion associated to the exposure of Cys61 to the solvent. This collective motion involves primarily the displacement of Cys61 without any other significant conformational changes (Fig 7 and S7 Fig).

According to our simulations, the protonation state of the Cys61 would be a switch to maintain this residue buried or exposed to the solvent. The transition between these two conformations must be fast once Cys61 is ionized. We analyzed the interactions that the Cys61 protonated residue establishes during our simulation of the free CsdE monomer with a neutral Cys61 and observed that the only residue capable of carry out its deprotonation would be Glu62 (S8 Fig), which in turn could transfer the proton to the bulk solvent. The driving force of the opening process would then be the proximity between two negatively charged residues, Cys61 and Glu62. The simulation of the free CsdE monomer with an anionic Cys61 shows that the distance between these two residues increases significantly in the solvent-exposed conformation (see S9 Fig and its correlation to Fig 6). According to this hypothesis, deprotonation of Cys61 would trigger the movement and exposure of this residue in the CsdE free form. This can be achieved before or after the formation of the CsdA-CsdE or CsdE-CsdE complexes and supports the proposal that the free CsdE monomer could exist in two different states *in vivo*. Alternatively, Cys61 deprotonation could be also carried out by another basic residue placed on the surface of the CsdA monomer, upon formation of the CsdE-CsdA complex. The more likely candidate is Arg353, whose side chain can be brought to within 2.5 Å of CsdE Cys61 thiol in the structure of persulfurated (CsdA-CsdE)_2_ by a change in rotamer conformation. We favor a conformational selection model for CsdA-CsdE complex formation in view of the dynamic conformational exchange that occurs spontaneously in solution and the experimentally determined p*K*_a_ for CsdE Cys61 thiol (6.5) [22].

### Functional relevance of Cys61 dynamic behavior

The strong sequence and structural resemblance between SufS-SufE and CsdA-CsdE provides the rationale for comparing findings on each sulfur donor-acceptor pair. Such comparisons can generate new insights and help build a greater appreciation for the molecular recognition events, conformational changes, and enzyme kinetics and mechanisms underpinning sulfur transfer across protein-protein interfaces. In contrast to the SufS-SufE interaction, which is inherently transient, CsdA and CsdE form a stable complex that can be purified as a heterotetramer and analyzed by biophysical and structural biology methods. The recent determination of two crystal structures of the (CsdA-CsdE)_2_ complex [20,21] indicates that the structural and functional analysis of the minimalistic CsdA-CsdE sulfur transfer system could provide insight into the SufS-SufE field. Likewise, the analysis of their distinct functional roles might shed light on the evolutionary conservation of both systems in *E*. *coli*.

A remarkable difference between the sulfur acceptors CsdE and SufE is found in their susceptibility to oxidation. Since the SUF system becomes upregulated upon oxidative stress or when iron is depleted, whereas the CSD system remains essentially constitutive, it is relevant to consider the relative stability of CsdE and SufE towards pro-oxidative factors. Indeed, SufE has been shown to be relatively resilient to the treatment with H_2_O_2_ owing to the fact that Cys51 remains stably secluded in a hydrophobic cage immediately below the SufE surface [39]. In contrast, CsdE readily forms disulfide-bridged dimers when exposed to oxidative environments. In fact, purifying CsdE without the addition of excess reducing chemical agents leads to the spontaneous formation of CsdE dimers [22]. Since the structure of free CsdE features a similarly secluded conformation for Cys61 both in solution by NMR [17] as in a recent crystal structure [19], we propose that the hydrophobic cage that buries Cys61 in CsdE is not as efficient as that in SufE. This proposal immediately suggests a mechanism for the oxidation of CsdE Cys61 by being exposed to solvent-dissolved oxygen a greater fraction of the time than Cys51 in SufE. Interestingly, this implies a greater intrinsic dynamism for CsdE Cys61 when compared with SufE Cys51. The crystal structure of a disulfide-bridged CsdE dimer, obtained from growing crystals out of pure CsdE monomers (attested by gel filtration and excess reducing agent in the protein stock), indicates that this spontaneous exposure of Cys61 is indeed a relatively common occurrence. Furthermore, the superposition of the three extreme case structures (free, oxidized dimer, and persulfurated CsdA-CsdE complex) delineates a conformational pathway that could be visited by Cys61. If this were the case, CsdE would lie in a metastable state where Cys61 could ping pong between a fully solvent-protected and a nearly fully exposed conformation with minimal structural changes, in particular without the costly structural rearrangements that occur at the α7 helix in the CsdA-CsdE complex. As indicated by the MD simulations, ionization of Cys61 could trigger the transition between the two conformations. The CsdE species that is most likely to bind CsdA could be the Cys61 solvent-exposed state, which would require the least conformational changes to fit into the relatively rigid active-site cavity of CsdA.

**Fig 8:**
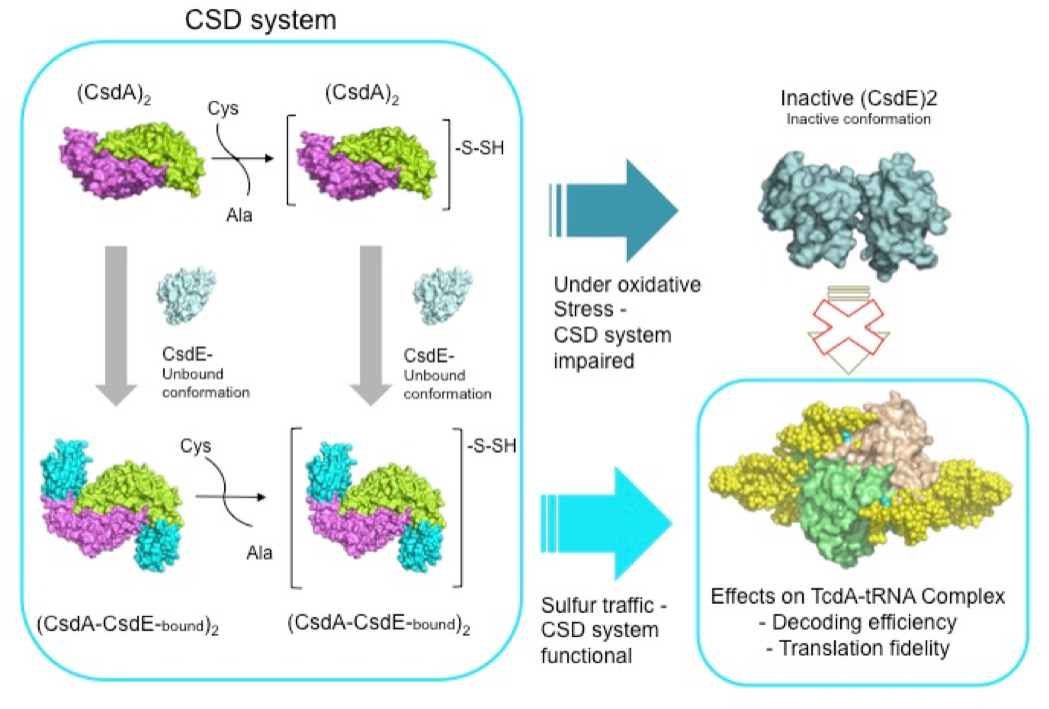
Functional consequences of CsdE inactivation by disulfide bridge formation. A functional CsdA-CsdE sulfur mobilization system is depicted inside a cyan outline. CsdA, CsdE, and TcdA are represented as molecular surfaces. The tRNA molecules in the TcdA-tRNA complex are bead models derived from SAXS [41]. CsdE is color coded in light cyan (CsdA unbound form) or bright cyan (bound to CsdA). CsdA subunits are colored in green and violet. TcdA subunits are shown in pale green and wheat. The tRNA bead models are in yellow. The inactivation of CsdE during oxidative stress conditions would likely lead to the impairment of the CsdA-CsdE downstream effector functions, the best known of which is the effect on ribosomal translation efficiency and fidelity through the TcdA-tRNA^ANN^ complex.

This strategy, which looks more efficient toward stimulating cysteine desulfurase activity, would come at the price of making the catalytic Cys61 more oxidation susceptible. Therefore, the preservation of Cys51 inside a fully buried conformation in SufE could be the result of evolutionary pressures aimed at conserving a reduced state for Cys51 through highly oxidative stress peaks, where CsdE Cys61 might become fully inactivated by disulfide bridging or *S*-oxidation. Under oxidative stress it is likely that CsdE becomes inactivated owing to the covalent linkage of CsdE monomers, thereby generating a cascade of molecular events that could impair downstream effectors such as TcdA and possibly other unknown effectors. In particular, the dependence of TcdA on a functional CsdA-CsdE pathway for the cyclization of t^6^A37 in the anti-codon stem loop (ASL) of tRNA^ANN^ in *E*. *coli* suggests that the oxidation-induced inactivation of CsdE could result in a *csdE* deletion-like phenotype (Fig 8) [15]. This phenotype is characterized by a marked decrease in ribosome decoding efficiency and fidelity and, in conjunction with other predisposing factors, could lead to severe cell growth defects. Ultimately, the exquisite redox sensitivity of sulfur acceptors such as CsdE underpin their potential for transmitting the deleterious effects of oxidative stress through the highly interconnected network of oxidation-sensitive sulfur donor and acceptor proteins.

## Supporting information

S1 Table Crystallographic data collection and refinement statistics. (PDF)

S2 Table Root-mean-square displacements (RMSD) between available CsdE structures. RMSD (in Å) calculated with the SSM algorithm. The upper triangular matrix reports the rmsd as a single value for one-to-one comparisons or, when multiple chains are present in the asymmetric unit of the corresponding structure, the average ± standard deviation (*N*, number of independent measurements). The diagonal (shaded) is assigned a value of 0.0 for one-to-one comparisons or the average ± standard deviation (*N*, number of independent measurements) for multiple comparisons. The lower triangular matrix reports the number of Cα atoms superimposed for rmsd computation. Cells containing data of comparisons with the dimeric CsdE structure (PDB 5nq6) are shaded in light green.
(PDF)

S3 Table Analysis of disulfide-bridged CsdE biological and crystallographic interfaces. Interface areas calculated with PISA v.2.0.7. Sym.Op: applies to 2nd monomer. Nhb: no. of hydrogen bonds. Nsb: no. of salt bridges. Nds: no. of disulfide bonds.
(PDF)

S1 Fig Root-mean-square deviations (RMSD, in Å) plotted along the simulation time (ns). (A) RMSD for the disulfide-bridged CsdE dimer. (B, C) RMSD for each of the monomers in the CsdE dimer.
(PDF)

S2 Fig Relative orientation between the two disulfide-linked CsdE monomers. CsdE is shown in ribbon representation, with each chain colored different (cyan and slate blue). The angle of 141.8° between the two monomers was calculated with the PyMOL script draw_symmetry_axis.py, and its spread and direction is visually shown by yellow CGO elements. The rotation axis runs exactly perpendicular to the plane of the figure, and is represented by an orange circle where the rotation axis and the plane intersect one another.
(PDF)

S3 Fig Dynamical evolution of the number of hydrogen bond contacts established between monomers of the CsdE dimer. (A) Number of bridge waters counted over the simulations. Bridge waters are at the same time hydrogen bonded to a residue of each monomer. (B) Direct hydrogen bonds between residues of each monomer. Average and standard deviation values are shown in each plot.
(PDF)

S4 Fig RMSD plots of the free CsdE monomers. Plot of free CsdE containing (A) a neutral Csy61 residue and (B) a Cys61 residue in its anionic state.
(PDF)

S5 Fig Computed and crystallographic *B*-factors from PDB 5eep. Molecular dynamics-derived *B*-factor values were determined from simulations of free CsdE monomers in solution with Cys61 in its neutral state (A) and in its anionic form (B). *B*-factors are expressed in Å^2^.
(PDF)

S6 Fig Pearson correlation coefficients between data and model for PLS-FMA. The correlation coefficient is plotted as a function of the number of PLS components calculated for the model training subset (R_m_) and validation subset (R_c_).
(PDF)

S7 Fig (Animation). **Conformational changes in CsdE leading to the exposure of the reactive Cys61 thiol**. Click on the image to visualize the animation (PowerPoint must be installed). Superimposition in cartoon with helices shown as cylinders CsdE free monomer with anionic Cys61 as obtained from the MD simulation (colored in green), the CsdE monomer of the disulfide-bridged dimer (colored in pale blue) and CsdE monomer from the X-Ray structure of the (CsdA-CsdE)_2_ complex (colored in cyan). The CsdE monomer is animated over the ensemble-weighted maximally correlated mode contributing to the change in the selected distance, *d*[Cys61Cα–Val88Cα]. Cys61 alpha carbon atom is shown as a yellow sphere.
(MP4)

S8 Fig Distance between the thiol hydrogen atom of Cys61 and the Oε of the Glu62 residue. (PDF)

S9 Fig Distance between the sulfur atom of Cys61 and the Cδ atom of the Glu62 residue. (PDF)
