# Supplementary material for: Insights into the inhibited form of the redox-sensitive SufE-like sulfur acceptor CsdE
Source: PLoS One. 2017 Oct 18;12(10):e0186286. doi: 10.1371/journal.pone.0186286 (PMC5646864; doi:10.1371/journal.pone.0186286)
Supplement: S1 Table — (PDF) [file pone.0186286.s001.pdf]

S1 Table. Crystallographic data collection and refinement statistics

		**Se-SAD (peak)**	**CsdE (PDB 5nq6)**
**Data collection**			
Diffraction source		ESRF ID23-1	ALBA BL13-XALOC
Wavelength (Å)		0.97885	0. 97949
Temperature (*K*)		100	100
Detector		ADSC Quantum 315 CCD	Dectris Pilatus 6M
Crystal-detector distance (mm)		415.9	601.8
Rotation range per image (º)		1.0	1.0
Total rotation range (º)		360.0	180.0
Exposure time per image (s)		0.5	1.0
Space group		$P\ 3_2\ 2$	$P\ 3_2\ 2$
Cell dimensions			
	a, *b*, *c* (Å)	58.53, 58.53, 153.42	58.43, 58.43, 150.45
	α, *β*, *γ* (º)	90, 90, 120	90, 90, 120
Mosaicity (º)		0.268	0.198
Resolution range (Å)		42.3−2.95	47.9−2.40
		(3.06−2.95)	(2.49−2.40)
Total No. of reflections		143,890 (14,015)	91,238 (3517)
No. of unique reflections		6880 (663)	12,185 (1143)
Completeness (%)		100 (100)	99.31 (96.05)
Anomalous Completeness (%)		100 (96.3)	
Redundancy		20.9 (21.1)	7.5 (3.1)
Anomalous Redundancy		11.4 (11.1)	
Mean I / σ(I)		25.69 (4.45)	13.45 (1.60)[a]
R_{merge}[b]		0.1574 (0.978)	0.0927 (0.711)
R_{meas}[c]		0.1614 (1.002)	0.0992 (0.853)
Overall *B*-factor from Wilson plot (Å^2)		57.89	44.91
$CC_{1/2}$[d]		0.999 (0.835)	0.999 (0.581)
CC*[e]		1.000 (0.954)	1.000 (0.857)
|DANO| / σ(DANO)		1.262 (0.807)	
**Refinement and validation**			
No. of reflections used in refinement			12,174 (1143)
No. of reflections used for R_{free}			583 (49)
R_{work}[f]			0.191 (0.264)
R_{free}[g]			0.248 (0.338)
No. of atoms			
	All (non-H)		2267
	Protein		2185
	Ligands		35
	Water		47
No. of residues (chains)			283 (2)
R.m.s. deviations			

	Bond lengths (Å)		0.008
	Bond angles (°)		1.02
B-factors ($Å^2$)			
	All		56.17
	Protein		56.00
	Ligands		70.13
	Water		53.34
Ramachandran plot			
	Favored (%)		98.58
	Allowed (%)		1.42
	Outliers (%)		0.00
Rotamer outliers (%)			0.00
Clashscore			10.05

Values for the higher resolution shell are given in parentheses.

[a]The maximum resolution of the native data set was chosen so that $CC_{1/2} > 0.5$ and the mean $I / \sigma(I) > 1.5$; the resolution at which the mean $I / \sigma(I)$ falls below 2.0 is 2.48 Å.

[b]$R_{merge} = \Sigma_{\mathbf{h}} \Sigma_i |I_i(\mathbf{h}) - \langle I(\mathbf{h})\rangle| / \Sigma_{\mathbf{h}} \Sigma_i I_i(\mathbf{h})$, where $\mathbf{h} = (hkl)$, $I_i(\mathbf{h})$ is the ith measurement and $\langle I(\mathbf{h})\rangle$ is the weighted mean of all measurements of $I(\mathbf{h})$

[c]$R_{meas} = \Sigma_{\mathbf{h}} (n / n - 1)^{1/2} \Sigma_i^n |I_i(\mathbf{h}) - \langle I(\mathbf{h})\rangle| / \Sigma_{\mathbf{h}} \Sigma_i I_i(\mathbf{h})$, where n is the number of independent observations of $I(\mathbf{h})$

[d]$CC_{1/2}$ is the Pearson correlation coefficient calculated between two random half data sets

[e]CC* is the CC of the full data set against the true intensities, estimated from $CC^* = [2\, CC_{1/2} / (1 + CC_{1/2})]^{1/2}$.

[f]$R_{work} = \Sigma_{\mathbf{h}} |F_O - F_C| / \Sigma_{\mathbf{h}} F_O$, where F_O and F_C are the observed and calculated structure factor amplitudes of reflection **h**

[g]R_{free} is as R_{work}, but calculated with 5% of randomly chosen reflections omitted from refinement
