# Supplementary material for: Insights into the inhibited form of the redox-sensitive SufE-like sulfur acceptor CsdE
Source: PLoS One. 2017 Oct 18;12(10):e0186286. doi: 10.1371/journal.pone.0186286 (PMC5646864; doi:10.1371/journal.pone.0186286)
Supplement: S2 Table — RMSD (in Å) calculated with the SSM algorithm. The upper triangular matrix reports the rmsd as a single value for one-to-one comparisons or, when multiple chains are present in the asymmetric unit of the corresponding structure, the average ± standard deviation (N, number of independent measurements). The diagonal (shaded) is assigned a value of 0.0 for one-to-one comparisons or the average ± standard deviation (N, number of independent measurements) for multiple comparisons. The lower triangular matrix reports the number of Cα atoms superimposed for rmsd computation. Cells containing data of comparisons with the dimeric CsdE structure (PDB 5nq6) are shaded in light green. (PDF) [file pone.0186286.s002.pdf]

S2 Table. Root-mean-square displacements (rmsd) between available CsdE structures, calculated with the SSM algorithm [1].

**rmsd / # of Cα atoms**	NMR CsdE (PDB ID 1ni7)	Xtal CsdE (PDB ID 5eep)	Xtal CsdA-CsdE (PDB ID 4lw4)	Xtal CsdA-CsdE (PDB ID 5ft8)	Xtal CsdE (PDB ID 5nq6)
NMR CsdE (PDB ID 1ni7)	0.0	1.56	1.5 ± 0.1 (2)	1.52 ± 0.08 (7)[a]	1.5 ± 0.2 (2)
Xtal CsdE (PDB ID 5eep)	139	0.0	1.10 ± 0.08 (2)	1.3 ± 0.1 (7)[a]	0.85 ± 0.03 (2)
Xtal CsdA-CsdE (PDB ID 4lw4)	129-131	131-135	0.85	0.8 ± 3 (14)<br>1.14 ± 0.09 (7)[b]<br>0.53 ± 0.04 (7)[b]	1.12 ± 0.04 (4)
Xtal CsdA-CsdE (PDB ID 5ft8)	127-136	132-136	130-137<br>130-137[b]<br>137[b]	0.4 ± 0.1 (21)[c]	1.3 ± 0.1 (14)
Xtal CsdE (PDB ID 5nq6)	137-139	139-140	131-135	129-137	1.10

The upper triangular matrix reports the rmsd as a single value for one-to-one comparisons or, when multiple chains are present in the asymmetric unit of the corresponding structure, the average ± standard deviation (*N*, number of independent measurements). The diagonal (shaded) is assigned a value of 0.0 for one-to-one comparisons or the average ± standard deviation (*N*, number of independent measurements) for multiple comparisons. The lower triangular matrix reports the number of Cα atoms superimposed for rmsd computation. Cells containing data of comparisons with the dimeric CsdE structure (PDB 5nq6) are shaded in light green.

[a] Only seven CsdE chains were superimposed for the comparisons because the eighth CsdE chain (chain P) is considerably more disordered than the others and its inclusion might distort the statistics.

[b] The comparison between chains C and D of the CsdA-CsdE complex published by Kim & Park [2] and all CsdE chains (except chain P) of the persulfurated CsdA-CsdE complex [3] showed a sharp contrast in conformation. While chain D and all persulfurated CsdE chains had rmsd’s tightly clustered around 0.5 Å for 135-137 Cα atoms, the corresponding rmsd for the same comparison with chain C was 1.14 Å, which is significantly greater.

[c] All seven chains of the persulfurated CsdA-CsdE complex were superimposed in pairs, therefore giving a total of 21 independent superpositions.

1. Krissinel E, Henrick K (2004) Secondary-structure matching (SSM), a new tool for fast protein structure alignment in three dimensions. Acta Crystallogr D Biol Crystallogr 60: 2256–2268. doi:10.1107/S0907444904026460.

2. Kim S, Park S (2013) Structural changes during cysteine desulfurase CsdA and sulfur acceptor CsdE interactions provide insight into the trans-persulfuration. J Biol Chem 288: 27172–27180. doi:10.1074/jbc.M113.480277.

3. Fernández FJ, Ardá A, López-Estepa M, Aranda J, Peña-Soler E, et al. (2016) Mechanism of Sulfur Transfer Across Protein–Protein Interfaces: The Cysteine Desulfurase Model System. ACS Catal 6: 3975–3984. doi:10.1021/acscatal.6b00360.
