# Supplementary material for: Insights into the inhibited form of the redox-sensitive SufE-like sulfur acceptor CsdE
Source: PLoS One. 2017 Oct 18;12(10):e0186286. doi: 10.1371/journal.pone.0186286 (PMC5646864; doi:10.1371/journal.pone.0186286)
Supplement: S3 Table — Interface areas calculated with PISA v.2.0.7. Sym.Op: applies to 2nd monomer. Nhb: no. of hydrogen bonds. Nsb: no. of salt bridges. Nds: no. of disulfide bonds. (PDF) [file pone.0186286.s003.pdf]

S3 Table. Analysis of disulfide-bridged CsdE biological and crystallographic interfaces, calculated with PISA v.2.0.7. [1]

Id	Mon.1	Mon.2	Sym. Operation	Sym.Id	Area (Å^2)	ΔG (kcal/mol)	Nhb	Nsb	Nds
1	A	A	Y-1,X+1,-Z+1	4_466	638.4	6.1	20	12	0
2	A	B	Y-1,X,-Z+1	4_456	619.5	-3.4	3	8	0
3	B	A	-Y,X-Y+1,Z-1/3	2_564	454.2	-7.2	6	0	0
4	B	B	-X-1,-X+Y-1,-Z+2/3	6_445	450.9	-0.9	6	2	0
5[a]	B	A	X,Y,Z	1_555	393.0	-3.9	2	0	0
6	A	A	Y-1,X,-Z+1	4_456	157.9	-2.1	0	0	0
7	B	B	-X,-X+Y,-Z+2/3	6_555	146.1	0.1	4	0	0
8	B	A	Y-1,X,-Z+1	4_456	16.0	0.7	0	0	0

Sym.Op: applies to 2nd monomer. Nhb: no. of hydrogen bonds. Nsb: no. of salt bridges. Nds: no. of disulfide bonds.

[a] The fifth row refers to the disulfide-bridged CsdE dimer interface.

1. Krissinel E (2011) Macromolecular complexes in crystals and solutions. Acta Crystallogr D Biol Crystallogr 67: 376–385. doi:10.1107/S0907444911007232.
