# Supplementary material for: Insights into the inhibited form of the redox-sensitive SufE-like sulfur acceptor CsdE
Source: PLoS One. 2017 Oct 18;12(10):e0186286. doi: 10.1371/journal.pone.0186286 (PMC5646864; doi:10.1371/journal.pone.0186286)
Supplement: S1 Fig — (A) RMSD for the disulfide-bridged CsdE dimer. (B, C) RMSD for each of the monomers in the CsdE dimer. (PDF) [file pone.0186286.s004.pdf]

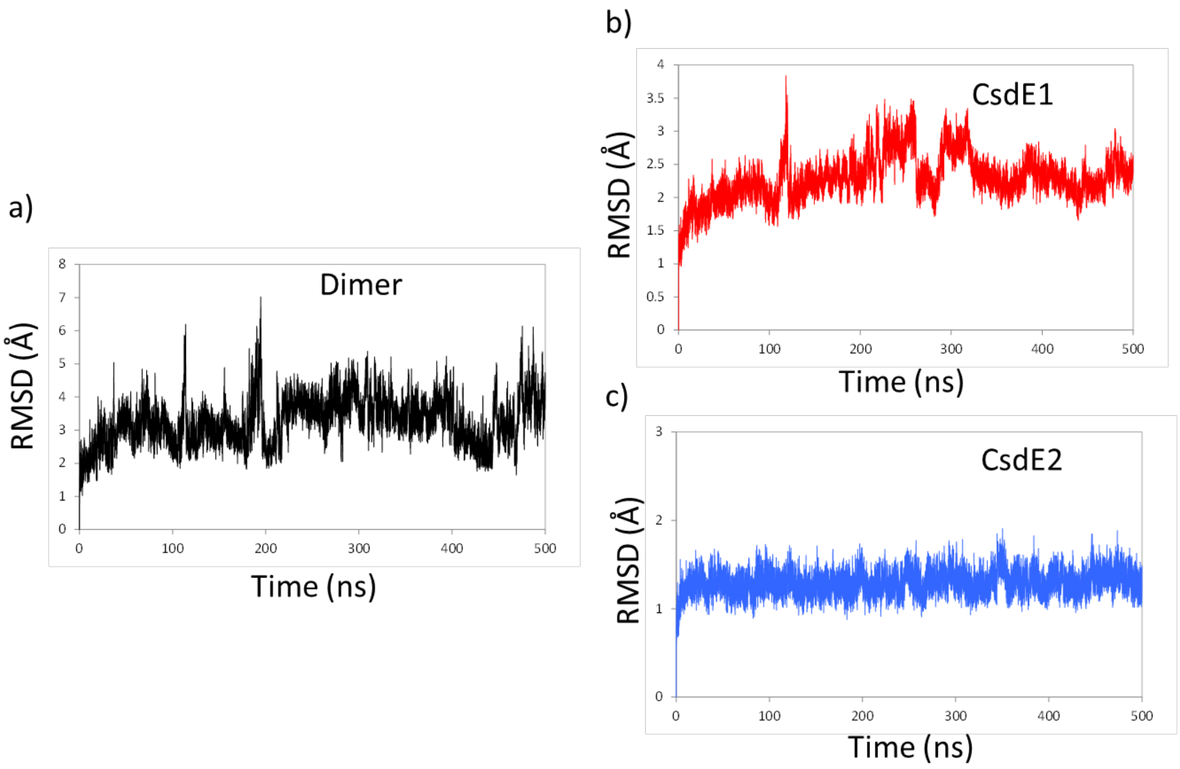

S1 Fig. Root-mean-square deviations (RMSD, in Å) for the disulfide-bridged CsdE dimer (a) and for each of the monomers ((b) and (c)) plotted along the simulation time (ns).
