# Supplementary material for: Insights into the inhibited form of the redox-sensitive SufE-like sulfur acceptor CsdE
Source: PLoS One. 2017 Oct 18;12(10):e0186286. doi: 10.1371/journal.pone.0186286 (PMC5646864; doi:10.1371/journal.pone.0186286)
Supplement: S2 Fig — CsdE is shown in ribbon representation, with each chain colored different (cyan and slate blue). The angle of 141.8° between the two monomers was calculated with the PyMOL script draw_symmetry_axis.py, and its spread and direction is visually shown by yellow CGO elements. The rotation axis runs exactly perpendicular to the plane of the figure, and is represented by an orange circle where the rotation axis and the plane intersect one another. (PDF) [file pone.0186286.s005.pdf]

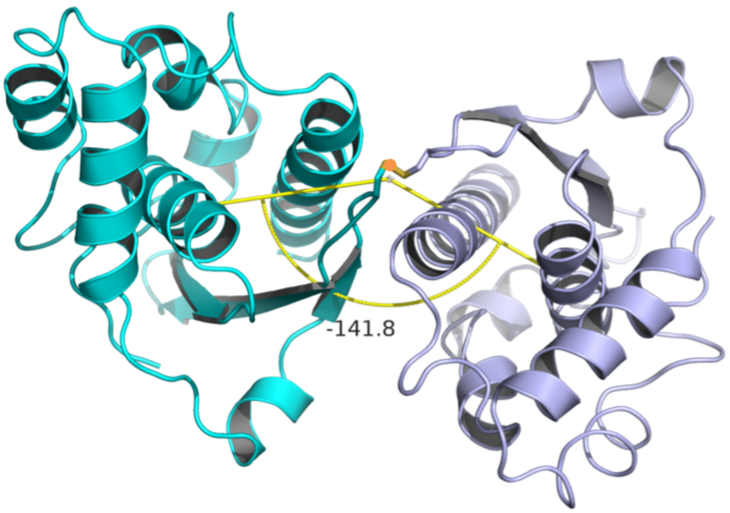

S2 Fig. Relative orientation between the two disulfide-linked CsdE monomers. CsdE is shown in ribbon representation, with each chain colored different (cyan and slate blue). The angle of 141.8º between the two monomers was calculated with the PyMOL script draw_symmetry_axis.py, and its spread and direction is visually shown by yellow CGO elements. The rotation axis runs exactly perpendicular to the plane of the figure, and is represented by an orange circle where the rotation axis and the plane intersect one another.
