# Supplementary material for: Insights into the inhibited form of the redox-sensitive SufE-like sulfur acceptor CsdE
Source: PLoS One. 2017 Oct 18;12(10):e0186286. doi: 10.1371/journal.pone.0186286 (PMC5646864; doi:10.1371/journal.pone.0186286)
Supplement: S3 Fig — (A) Number of bridge waters counted over the simulations. Bridge waters are at the same time hydrogen bonded to a residue of each monomer. (B) Direct hydrogen bonds between residues of each monomer. Average and standard deviation values are shown in each plot. (PDF) [file pone.0186286.s006.pdf]

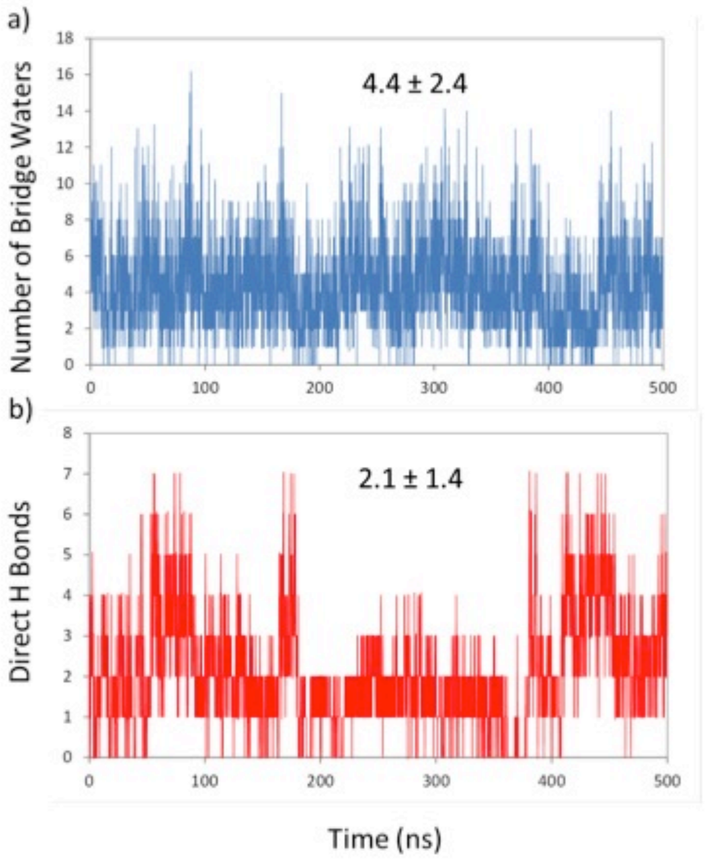

S3 Fig. **Dynamical evolution of the number of hydrogen bond contacts established between monomers of the CsdE dimer.** (a) the number of bridge waters counted over the simulations. Bridge waters are at the same time hydrogen bonded to a residue of each monomer. (b) Direct hydrogen bonds between residues of each monomer. Average and standard deviation values are shown in each plot.
