# Supplementary material for: Insights into the inhibited form of the redox-sensitive SufE-like sulfur acceptor CsdE
Source: PLoS One. 2017 Oct 18;12(10):e0186286. doi: 10.1371/journal.pone.0186286 (PMC5646864; doi:10.1371/journal.pone.0186286)
Supplement: S4 Fig — Plot of free CsdE containing (A) a neutral Csy61 residue and (B) a Cys61 residue in its anionic state. (PDF) [file pone.0186286.s007.pdf]

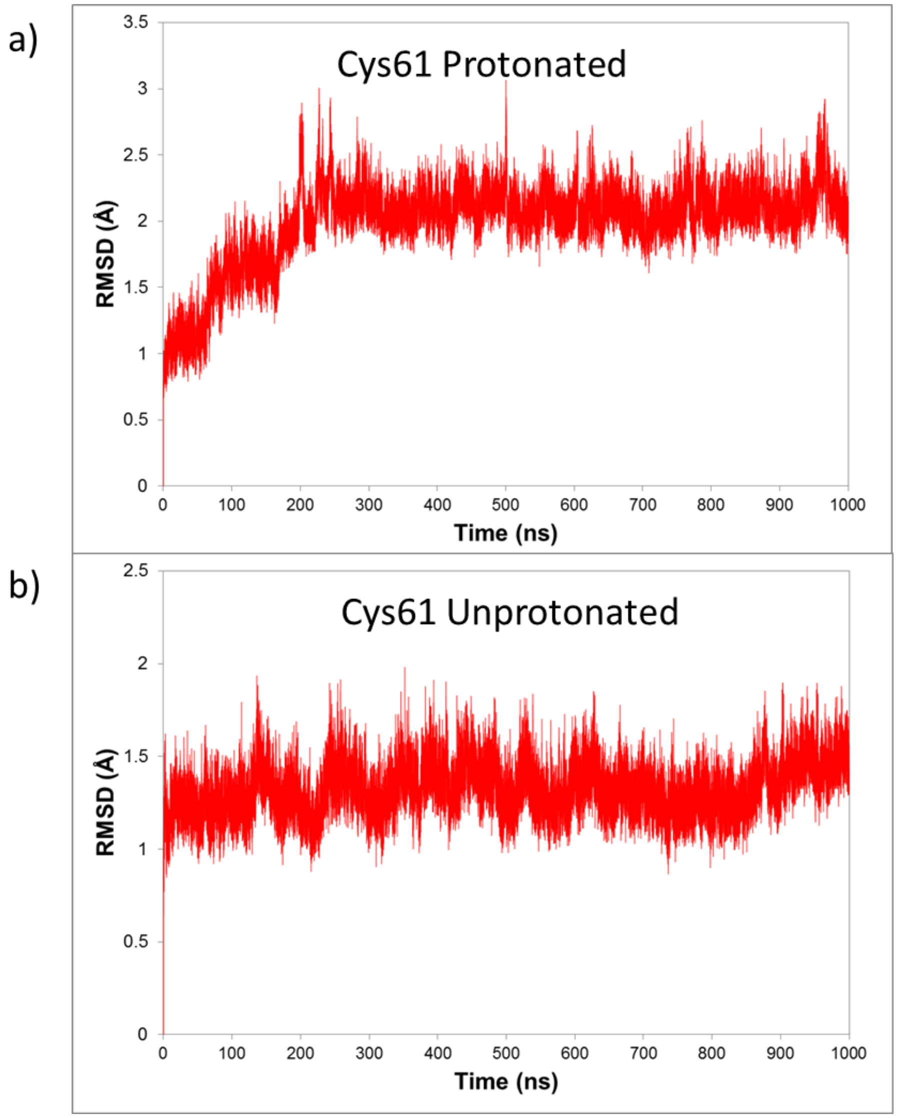

S4 Fig. RMSD plots of the free CsdE monomers. Plot of free CsdE containing (a) a neutral Csy61 residue and (b) a Cys61 residue in its anionic state.
