# Supplementary material for: Insights into the inhibited form of the redox-sensitive SufE-like sulfur acceptor CsdE
Source: PLoS One. 2017 Oct 18;12(10):e0186286. doi: 10.1371/journal.pone.0186286 (PMC5646864; doi:10.1371/journal.pone.0186286)
Supplement: S5 Fig — Molecular dynamics-derived B-factor values were determined from simulations of free CsdE monomers in solution with Cys61 in its neutral state (A) and in its anionic form (B). B-factors are expressed in Å2. (PDF) [file pone.0186286.s008.pdf]

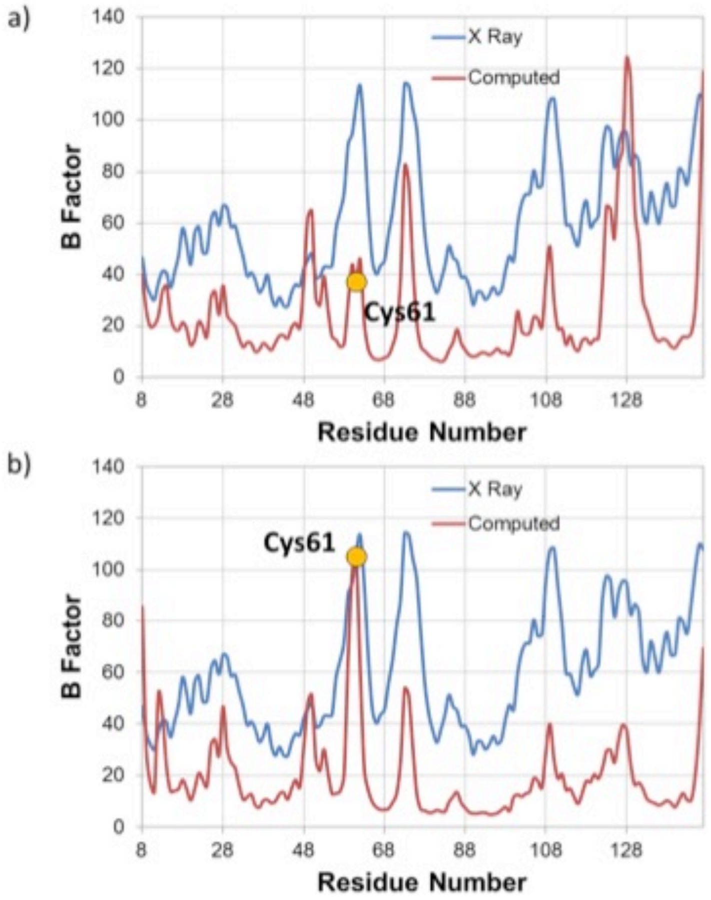

S5 Fig. **Computed and crystallographic *B*-factors from PDB 5eep.** Molecular dynamics-derived *B*-factor values were determined from simulations of free CsdE monomers in solution with Cys61 in its neutral state (a) and in its anionic form (b). *B*-factors are expressed in $Å^2$.
