# Supplementary material for: Insights into the inhibited form of the redox-sensitive SufE-like sulfur acceptor CsdE
Source: PLoS One. 2017 Oct 18;12(10):e0186286. doi: 10.1371/journal.pone.0186286 (PMC5646864; doi:10.1371/journal.pone.0186286)
Supplement: S6 Fig — The correlation coefficient is plotted as a function of the number of PLS components calculated for the model training subset (Rm) and validation subset (Rc). (PDF) [file pone.0186286.s009.pdf]

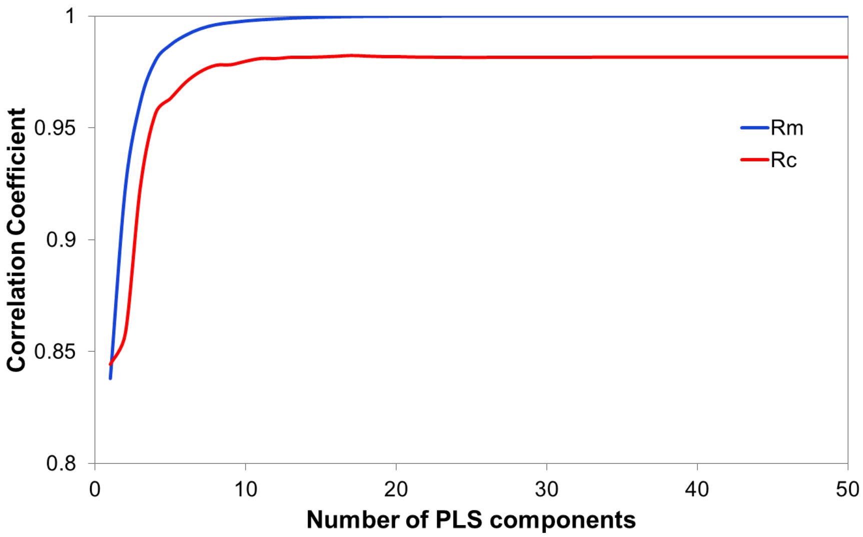

S6 Fig. Pearson correlation coefficients between data and model for PLS-FMA. The correlation coefficient is plotted as a function of the number of PLS components calculated for the model training subset (R_m) and validation subset (R_c).
