# Supplementary figures and images for: Insights into the inhibited form of the redox-sensitive SufE-like sulfur acceptor CsdE
Source: PLoS One. 2017 Oct 18;12(10):e0186286. doi: 10.1371/journal.pone.0186286 (PMC5646864; doi:10.1371/journal.pone.0186286)

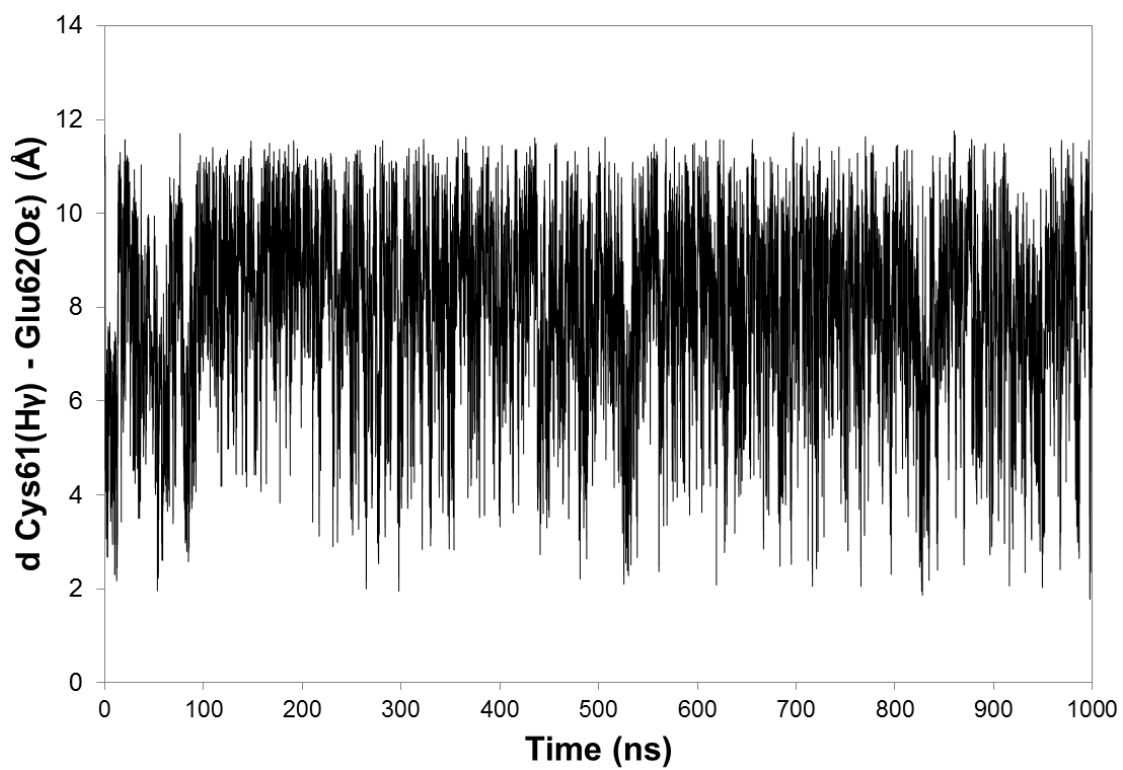

**S8 Fig.** Distance between the thiol hydrogen atom of Cys61 and the O $\epsilon$  of the Glu62 residue.

Supplement: S8 Fig — (PDF) [file pone.0186286.s011.pdf]

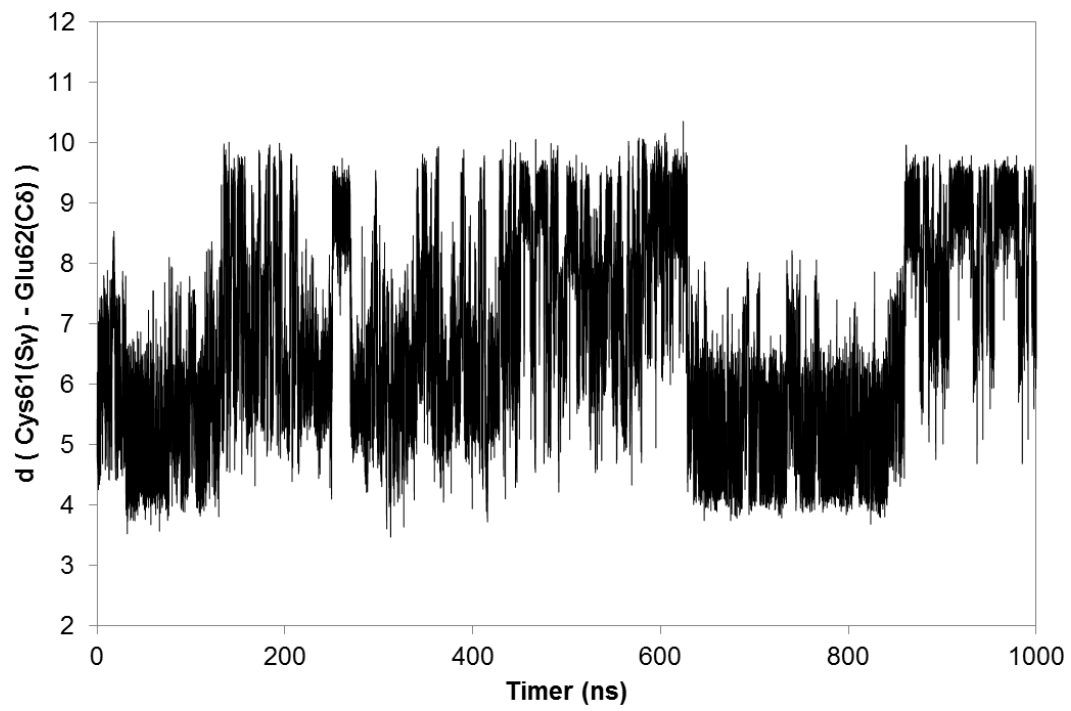

**S9 Fig.** Distance between the sulfur atom of Cys61 and the C $\delta$  atom of the Glu62 residue.

Supplement: S9 Fig — (PDF) [file pone.0186286.s012.pdf]
